# Impact of a stakeholder selected implementation strategy package – fast tracking, provider re-training, and co-location – on PrEP implementation for pregnant women in antenatal care clinics in western Kenya

**DOI:** 10.1186/s43058-025-00746-5

**Published:** 2025-05-12

**Authors:** Joseph Sila, Anjuli Dawn Wagner, Felix Abuna, Julia C Dettinger, Ben Odhiambo, Nancy Ngumbau, George Oketch, Enock Sifuna, Laurén Gómez, Sarah Hicks, Bryan J. Weiner, Grace John-Stewart, John Kinuthia

**Affiliations:** 1 Research & Programs, Kenyatta National Hospital, Nairobi, Kenya; 2 Department of Global Health, University of Washington, Seattle, WA USA; 3 Department of Epidemiology, University of Washington, Seattle, WA USA; 4 Health Systems and Population Health, University of Washington, Seattle, WA USA; 5 Departments of Pediatrics & Medicine, University of Washington, Seattle, WA USA

**Keywords:** Pre-exposure prophylaxis (PrEP), Pregnancy, Postpartum, Implementation science, Integration, Fast tracking, Training, Co-location

## Abstract

**Background:**

Pre-exposure prophylaxis (PrEP) is recommended for HIV prevention in pregnant and postpartum women at substantial ongoing risk for HIV. In resource-limited settings, there exist gaps in the integration of PrEP into antenatal care.

**Methods:**

We conducted a difference-in-differences analytic approach (3 months pre- and 3 months post) between January 2022 and July 2022 in 8 facilities (4 intervention and 4 comparison) in western Kenya. During the 6-month period, we tested a combination of 2 stakeholder selected implementation strategies – retraining health providers and fast tracking PrEP clients– to improve PrEP delivery. All study facilities dispensed PrEP in the Maternal and Child health clinics (MCH). We evaluated absolute changes in: PrEP penetration, PrEP fidelity, client PrEP knowledge, client satisfaction, and client waiting and service times as outcomes specified a priori while PrEP offer and HIV testing were outcomes specified post hoc. We measured acceptability and appropriateness by providers of the implementation strategies using AIM and IAM respectively.

**Results:**

We observed statistically significant improvements in PrEP penetration and PrEP offer (*p* < 0.05) and non-significant improvements in fidelity. PrEP penetration increased 6 percent points (*p* = 0.002), PrEP offer increased nearly 6 percentage points (*p* = 0.002), and PrEP fidelity increased 4 percentage points (*p* = 0.202) in intervention vs comparison facilities. Client PrEP knowledge increased 0.45 out of 7 total points (*p* < 0.001) and PrEP screening increased 13 percentage points (*p* = 0.001). We observed no significant changes in service time (0.13-min increase; *p* = 0.249), waiting time (0.03-min decrease; *p* = 0.796), or client satisfaction (0.04/24 total point decrease; *p* = 0.849) in intervention vs comparison facilities. HIV testing did not significantly change (7 percentage point decrease, *p* = 0.305). The implementation strategy bundle was deemed appropriate and acceptable by the providers (appropriateness: 18.5/20; acceptability: 18.5/20). Overall, the implementation strategy bundle was associated with larger increases in implementation outcomes among women receiving a visit other than their first ANC visit, as well as among sites without stockouts of HIV test kits.

**Conclusions:**

A stakeholder-selected implementation strategy bundle that included retraining healthcare workers, fast tracking PrEP clients to reduce waiting time, and PrEP dispensing in MCH improved several implementation outcomes without significantly affecting waiting time or reducing service time.

**Supplementary Information:**

The online version contains supplementary material available at 10.1186/s43058-025-00746-5.

Contributions to the literature

- Evidence is sparse on effective implementation strategies that focus on structural- and provider-level barriers to enhance delivery of integrated care in resource-limited settings.
- Stakeholder-selected strategies are hypothesized to have a better fit to local context and potentially be more effective than externally selected strategies.
- We tested a package of two stakeholder-selected implementation strategies – retraining health providers, fast tracking PrEP clients in a difference-in-differences study in 8 clinics in Kenya.
- The package was associated with significant improvements in PrEP penetration, PrEP offer, PrEP knowledge, and PrEP screening. It was not associated with significant changes in PrEP fidelity, HIV testing, service time, waiting time, or client satisfaction.
- The improvement associated with the package was most pronounced among clinics that did not experience substantial stockouts of supplies, suggesting that implementation strategies are not sufficient to overcome gaps in basic resourcing in the presence of critical events.

## Introduction

HIV acquisition is high during pregnancy and postpartum and contributes disproportionately to new infant HIV infections [1–3]. Pre-exposure prophylaxis (PrEP) is safe, effective, and recommended by the World Health Organization (WHO) for HIV prevention [4–7]. PrEP for pregnant and postpartum populations can be delivered feasibly and acceptably within maternal and child health (MCH) clinics. MCH clinics in Kenya provide antenatal care, postnatal care, child welfare (child immunization, well-baby checks, growth monitoring), and curative services. Such integrated PrEP delivery models take advantage of high attendance at antenatal care, have less stigma than offering HIV prevention services within HIV care clinics, and have been found to be preferable to women in formative work [8].

The HIV prevalence in Kenya is estimated at 3.2% (UNAIDs 2023 estimates) with women disproportionately having higher prevalence than men (4.3% vs 2.1%) [9]. According to the Kenya’s guidelines for HIV prevention and treatment 2022 [10], HIV negative women at substantial risk of getting HIV are eligible for PrEP and should be offered PrEP. Integrated PrEP delivery in MCH has been previously tested in Kenya in research and demonstration project settings [8, 11–19]. Overall, PrEP uptake is higher in contexts with added staff responsible for PrEP delivery steps; PrEP uptake in two large Kenyan projects with added staff was 19% and 22% [11, 13], while uptake was 4% in two additional Kenyan studies in similar geographic areas in which added staff were unavailable [18, 20]. This study builds on the lessons from the two studies (PrIYA project and PrIMA study (NCT 03070600)), which evaluated models for integrated PrEP delivery within MCH clinics in western Kenya [11, 13]. A systematic review in 2020 of implementation science focused on PrEP in pregnancy and postpartum identified that most implementation strategies intervened at the intra- and interpersonal levels, rather than focusing on strategies that acted at the systems level [21]. While PrIYA and PrIMA included dedicated study staff, this study assessed strategies to address gaps observed after the end of research funding using existing facility staff. Human resource shortages are common in resource-limited settings; identifying implementation strategies to improve integrated PrEP delivery in the absence of added staff are critical.

Stakeholder-derived implementation strategies may be preferable to researcher-selected strategies due to improved fit with the context, selection being driven by stakeholder-informed perceptions of feasibility, and more detailed specification. In this study, we tested a combination of stakeholder selected strategies *– as previously described in Hicks *et al. [22] *–* to improve the implementation of PrEP delivery integrated into MCH clinics in Kenya. This study was part of a series of four tests of implementation strategy bundles [20].

## Methods

### Design and setting

This difference-in-difference study was conducted between January 2022 and July 2022 at eight facilities (4 intervention and 4 comparison) in Kisumu, Siaya, and Homa Bay counties in Western Kenya (Supplementary Table 1). We included 3 months of baseline time, during which no clinics received any implementation strategies, and 3 months of intervention time, during which we piloted an implementation strategy package in the 4 intervention clinics but not the 4 comparison clinics. The study was registered at ClinicalTrials.gov (NCT04712994).

Participant recruitment: Data was collected from several sources, including anonymous exit surveys from women seeking MCH services, surveys with health care providers, and abstraction from facility registers between January 17, 2022, and July 6, 2022 (Table 1). All women seeking MCH services at the study facilities who were > 15 years (including emancipated minors) and able to provide oral consent were eligible to participate in the study. Trained study nurses obtained oral consent and administered a client exit survey which assessed participant demographics, HIV risk screening and counseling, PrEP knowledge and client satisfaction with services received that day using REDCap. Women who agreed to participate in the time and motion survey carried a time and motion card, where HCWs recorded the time in and time out for each service received.

**Table 1:** Difference in differences comparison of implementation, effectiveness, and service outcomes

*Outcome*	*Definition*	Comparison sites				Intervention sites				Difference in difference [(Change in intervention sites) – (Change in comparison sites)] ***Adjusted for ANC		
		Pre (*N* = 420)		Post (*N* = 416)		Pre (*N* = 392)		Post (*N* = 408)		Point estimate	Confidence interval	*p*-value
		N	n (%) or median IQR or Mean (SD)	N	n (%) or median IQR or Mean (SD)	N	n (%) or median IQR or Mean (SD)	N	n (%) or median IQR or Mean (SD)			
PrEP fidelity^1^	**Proportion of women who receive all PrEP specific steps in a visit HIV testing, HIV risk screening, PrEP counseling/total women receiving antenatal or postnatal services (Risk screening question:"Please answer yes if you were asked about any of the following behaviors today: [Kenyan risk assessment tool HIV risk factors]")	163	8 (4.9%)	159	2 (1.3%)	179	9 (5.0%)	148	9 (6.1%)	4.1%	(−2.2%—10.3%)	0.202
HIV testing ^3^	**Proportion of women HIV tested among eligible (not tested in past 6 months, not known HIV positive)	164	94 (57.3%)	159	70 (44.0%)	180	93 (51.7%)	150	63 (42.0%)	−6.6%	(−19.2%—6.0%)	0.305
PrEP risk screening ^3^	**Proportion asked questions on HIV risk behavior characteristics (yes vs no/don’t know)	420	101 (24.0%)	416	87 (20.9%)	392	97 (24.7%)	408	146 (35.8%)	13.2%	(5.4%—21.0%)	0.001
PrEP penetration ^1^	**Proportion of women who are counseled about PrEP/total women receiving antenatal or postnatal services ("Did anyone talk to you about PrEP today?")	420	23 (5.5%)	416	7 (1.7%)	391	13 (3.3%)	408	23 (5.6%)	6.0%	(2.2%—9.8%)	0.002
PrEP offer ^3^	**Proportion offered to start or continue taking PrEP/total women receiving antenatal or postnatal services	419	24 (5.7%)	415	7 (1.7%)	392	12 (3.1%)	408	20 (4.9%)	5.8%	(2.1%—9.5%)	0.002
	**Proportion offered to start or continue taking PrEP (among those who were HIV negative and at high risk who were screened)	42	4 (9.5%)	31	1 (3.2%)	39	7 (18.0%)	23	8 (34.8%)	–	–	–
PrEP uptake^2^	**Proportion initiated PrEP today (among those offered)	24	2 (8%)	7	3 (43%)	12	2 (17%)	20	2 (10%)	–	–	–
	**Proportion initiated PrEP today (among total women receiving antenatal or postnatal services)	420	2 (0.5%)	416	3 (0.7%)	392	2 (0.5%)	408	2 (0.5%)	–	–	–
PrEP continuation ^2^	**Already taking PrEP and will continue to take PrEP (among those offered)	24	6 (25.0%)	7	1 (14.3%)	12	2 (16.7%)	20	6 (30%)	–	–	–
	Already taking PrEP and will continue to take PrEP (among total women receiving antenatal or postnatal services)	420	7 (1.7%)	416	3 (0.7%)	392	7 (1.8%)	408	8 (2.0%)	–	–	–
Service time ^1^	***Number of minutes receiving services from health care workers	192	14 (9.5—26.5)	192	12 (8.5—21.5)	192	15 (10—29)	192	15 (10—31.5)	0.13	(−0.09—0.35)	0.249
Waiting time ^1^	***Number of minutes spent waiting to receive services	192	33 (13.5—57.5)	192	38 (14.5—73)	192	47 (22—77.5)	192	42.5 (28—64)	−0.03	(−0.26—0.20)	0.796
Client satisfaction ^1^	**Satisfaction on a scale of 0–24 points; 6 questions of clients to assess their satisfaction with services received at the facility; Likert scale (worst to best: 1–5)	420	22.0 (20.0, 23.0)	416	21.0 (20.0, 22.0)	392	22.0 (20.0, 23.0)	408	21.0 (20.0, 22.0)	−0.04	(−0.40—0.33)	0.849
HCW satisfaction ^1^	****Average on 4 item Intervention Appropriateness Measure (IAM) scale; Likert scale (disagree to agree: 1–5)		–		–		–	64	18.5 (16.0—20.0)	–	–	–
	****Average on 4 item Acceptability of Intervention Measures (AIM) scale; Likert scale (disagree to agree: 1–5)		–		–		–	64	18.5 (16.0, 20.0)	–	–	–
Client PrEP knowledge ^2^	**All correct answers (6 questions based on content covered in counseling sessions)	420	6 (1.4%)	416	1 (0.2%)	392	3 (0.8%)	408	3 (0.7%)	1.2%	(−0.5%—2.8%)	0.181
	Number of correct answers (0–6)	420	1.13 (1.5)	416	0.77 (1.2)	392	0.94 (1.4)	408	1.02 (1.4)	0.45	(0.18—0.71)	< 0.001

^*^not adjusted for visit type due to visit type not collected during time-and-motion activity

^1^Primary outcome

^2^Secondary outcome

^3^Post hoc outcome

Data sources:

^**^Exit surveys with women receiving antenatal or postnatal services

^***^time and motion observations

^****^endline surveys with healthcare workers (HCWs)

### Selection and development of strategy bundle

As previously described [22], we conducted a multi-stage process with a variety of stakeholders to select a series of bundles of implementation strategies to test. Briefly, we first conducted qualitative focus groups to assess the range of implementation strategies tried organically by frontline healthcare workers with experience delivering integrated PrEP [23]. Next, we conducted quantitative surveys with experienced healthcare workers to assess whether they had tried each of the 16 implementation strategies and whether they improved PrEP delivery [22]. Next, we conducted a stakeholder workshop with PrEP stakeholders from national and county levels, facility in-charges, implementing partners, health care providers, and women receiving PrEP services. Stakeholders completed relative rankings of the strategies, then completed small group discussions and rated strategies in terms of perceived feasibility and effectiveness, creating “go-zone” quadrant plots, and then re-ranked the strategies following discussion of the most promising strategies. The study team then combined these rankings and ratings and bundled together three strategy bundles for piloting in MCH clinics. The first implementation strategy bundle contained 2 components: 1) fast-tracking PrEP clients to minimize waiting time, and 2) retraining PrEP providers [22, 24]. Below, we operationalize each strategy as per Proctor et al.’s implementation strategy specification [25].

### Fast tracking PrEP clients

While fast-tracking was highlighted as a promising strategy during qualitative and quantitative formative work [22, 23], it was more challenging to create an implementation plan at facilities to operationalize fast-tracking. It was not deemed acceptable to have women who were initiating PrEP to skip the queue; however, having women who were refilling PrEP skip the queue was deemed acceptable and possibly could improve efficiency. This study tested this operationalization of fast-tracking for clients seeking PrEP refills, rather than one that would prioritize clients initiating PrEP. In order to make the fast-tracked visits efficient, clinicians prepared PrEP prescriptions each day based on the visit schedule diary. We hypothesized that this strategy would reduce client waiting time, increase provider–client interaction, and enhance privacy.

### PrEP providers re-training

With the help of facility in-charges, we identified all HCWs involved in PrEP delivery activities at both MCH and HIV care clinics. Health care providers in intervention sites attended a two-day training on PrEP using the PrEP training curriculum from the National AIDS and STI Control Program (NASCOP) of the Ministry of Health in Kenya. Participants included nurses, HIV testing providers, peer educators, clinicians, health records officers and mentor mothers. The training was facilitated by study coordinators with support from County AIDS and STI Coordinators (CASCOs) and covered the following modules: background and rationale for PrEP, PrEP counseling, PrEP initiation, follow-up, restart and discontinuation, values clarification, patient-centered communication skills, referral and linkages for PrEP services, monitoring and evaluation for PrEP, and the wheel of behavior change. The training included brainstorming, case studies and case scenarios, interactive lectures, demonstrations and role plays, plenary discussions with question-and-answer sessions, group work and discussions, self-awareness exercises, and experience sharing. The HCWs in groups discussed myths and misconceptions about PrEP in addition to barriers and facilitators and how to tackle them. They also discussed case scenarios on risk assessment, PrEP counseling, PrEP initiation, and follow up. We hypothesized that this strategy would increase provider competence, translating to increased PrEP fidelity and penetration, as well as client knowledge.

### Dispensing PrEP in MCH clinics

Prior studies in Kenya have demonstrated high feasibility and acceptability of dispensing PrEP in MCH clinics instead of referring MCH clients to a central pharmacy or an HIV care specific pharmacy [11, 13]. All the study facilities dispensed the PrEP medication in either MCH clinics or the MCH pharmacy. We hypothesized that this strategy would reduce client waiting time, reduce stigma, and increase PrEP fidelity and uptake.

### Implementation, service and client outcomes

We measured implementation outcomes, service, and client outcomes as per Proctor et al. [26]. Our primary implementation outcomes (previously described in [20]) included PrEP penetration (proportion of women talked to about PrEP), PrEP fidelity (proportion of women who receive all PrEP specific steps in a visit: HIV testing, HIV risk screening, PrEP counseling), strategy bundle acceptability and appropriateness by HCWs, and PrEP uptake. We measured service outcomes, client waiting and service times, and client outcomes through client satisfaction. We assessed client PrEP knowledge as our secondary outcome, while PrEP offer (Proportion offered to start or continue taking PrEP) and HIV testing were assessed as post hoc outcomes (Table 1). Different components of PrEP knowledge assessed included: PrEP for HIV prevention, frequency of use, time to reach maximum protection, condom use while on PrEP, side effects, and discontinuation.

### Health care providers feedback

HCWs from the intervention facilities evaluated the appropriateness and acceptability of the strategy bundle tested using the Acceptability of Intervention Measure (AIM), Intervention Appropriateness Measure (IAM) [27]. The providers’ feedback included HCWs offering services in the MCH clinics, such as nurses, HIV testing providers, clinicians, peer counsellors and mentor mothers. We approached HCWs who participated in testing the strategy bundle in MCH clinics to complete a REDCap survey online. The study staff guided the HCWs through oral consent and once consented, they received an email or short message services link to the survey which they were given 2 weeks to complete. Additional 2 weeks and 2 follow up phone calls attempts one week apart were given to HCWs who did not complete the survey after which they were excluded and counted as lost to follow up.

### Ethical approval

The University of Washington Institutional Review Board and Kenyatta National Hospital/University of Nairobi Ethics & Research Committee approved this study under STUDY00008392 and P907/11/2019 approval numbers. To conduct the study in the facilities, the department of health from 3 counties approved the study. All participants provided oral consent before participating in the study.

### Statistical analysis

Descriptive statistics were used to summarize the participant characteristics in baseline and intervention periods. Categorical variables were summarized as numbers and percentages while continuous variables as means and standard deviations or medians and interquartile ranges. Changes associated with the implementation strategy bundle were measured using a difference-in-difference approach and multi-level mixed-effect regression model with a random effect for site. We used a binary term for intervention vs comparison group and for pre/post period and an interaction term between the two. Changes related to implementation strategy package were estimated as interaction terms, and changes were considered statistically significant at alpha ≤ 0.05. Percentage point changes were reported to reflect absolute, rather than relative differences in outcomes over time. All analyses were adjusted for whether women were seeking first ANC visits versus any other type of service to address confounding. We conducted sensitivity analyses in which we stratified by whether it was a woman’s first antenatal care visit versus any other type of visit.

In order to assess possible health systems improvement potential, we conducted a hypothetical best case scenario analysis to estimate the hypothetical number of women who would have been expected to have initiated PrEP if PrEP risk assessment, HIV testing, and PrEP offer were perfectly delivered but without changes to the proportion of women who initiated PrEP among those offered. As previously described [20], we multiplied the total number of women in our study by the proportion who would have had a risk indication had they been screened for PrEP, by the proportion who would have been eligible for HIV testing, by the proportion who would have tested HIV negative, by the proportion who would have accepted PrEP if offered (proportions based in the observed data in this study). We compared this hypothetical expected number of PrEP initiations under perfect conditions to the observed number of PrEP initiations in this study in a ratio. Analyses were performed using STATA version 18.0.

### Situational factors

**Fig. 1:**
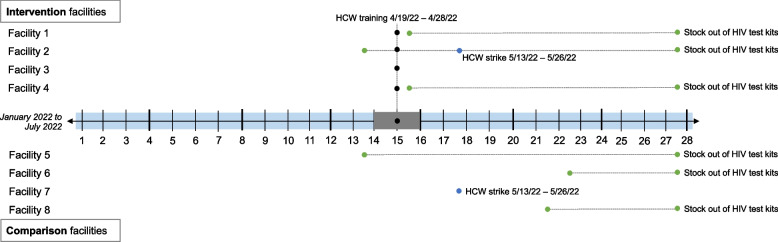
Timeline of service disruptions

A nearly countrywide HIV test kit stockout affected the delivery of PrEP during the intervention period. HCW strikes in Homa Bay county affected not only PrEP implementation but also other service delivery. Other events/activities that affected service delivery either during the 3-months of pre-intervention and 3 months during intervention are shown in Fig. 1. We conducted sensitivity analyses in which we stratified by whether a site experienced multiple months of HIV test kit stockout (6 facilities, 3 in each of the intervention and comparison) versus no stockouts of HIV test kits (2 facilities, 1 in each of the intervention and comparison).

## Results

### Participant characteristics

A total of 1,636 women receiving MCH services participated in a difference-in-differences study conducted in 8 facilities in western Kenya. Of the participants, 15.9% were seeking first ANC services and the overall median age was 25 (interquartile range [IQR]: 21, 29) years (Supplementary Table 2). There were differences between the number of first ANC women seeking services in comparison and intervention sites between periods (comparison sites: pre: 20.0%, post: 13.2%; intervention: pre: 17.9%, post: 12.5%) (Supplementary Table 2); all comparative analyses included adjustment for this difference. Sixty-four (64) HCWs participated in evaluating the acceptability and appropriateness of the strategies tested. The median age of HCW was 32 (IQR: 29–40) years, 73% female and 85% having polytechnic/college education and above. The median years of experience in providing services to pregnant and breastfeeding women was 5.4 (IQR: 2.2–7.5) years.

### Implementation outcomes

**Fig. 2:**
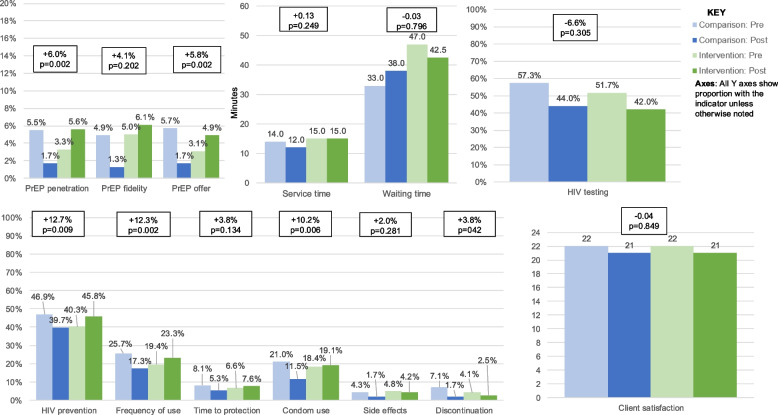
Implementation and service outcomes associated with implementation strategy package

Among comparison and intervention facilities during the baseline period, PrEP penetration ranged from 1.0–15.7%, PrEP fidelity from 0–15.8%, PrEP offer among eligible women from 1.0–15.7%, and HIV testing from 35%−81% (Supplementary Table 3). Due to prolonged stockouts of HIV testing kits across the region during the post period, there were substantial decreases in PrEP delivery at comparison facilities; PrEP penetration (5.5% during pre; 1.7% during post), PrEP fidelity (4.9% vs 1.3%), PrEP offer (5.7% vs 1.7%), and HIV testing (57.3% vs 44.0%). Waiting time was longer (33 vs 38 min), service time was shorter (14 vs 12 min), and PrEP knowledge decreased (1.4% vs 0.2%) (Table 1, Fig. 2).

Comparing intervention to comparison sites using a difference-in-differences approach, the implementation strategy bundle was associated with significant improvements in PrEP penetration and PrEP offer but not in PrEP fidelity or HIV testing. PrEP penetration was 6.0% percentage points (95%CI: 2.2%−9.8%; *p* = 0.002) greater in intervention sites compared to comparison sites. There was a significant and substantial increase in PrEP offer by 5.8% percentage points (95%CI: 2.1%−9.5%; *p* = 0.002) in intervention sites vs comparison sites. There was a non-significant increase in PrEP fidelity by 4.1% percentage points (95%CI: −2.2%−10.3%; *p* = 0.202) and non-significant decrease by 6.6% percentage points (95%CI: −19.2%−6.0%; *p* = 0.305) in HIV testing in intervention sites vs comparison sites. HIV risk screening significantly increased by 13.2% percentage points (95%CI: 5.4%−21.0%; *p* = 0.001) in intervention sites compared to comparison sites (Table 1, Fig. 2). PrEP uptake had low counts overall, which made statistical comparison inappropriate and not possible.

The implementation strategy bundle was highly rated by HCW on acceptability and appropriateness. The median acceptability score was 18.5 (IQR: 16.0–20.0) while the median appropriateness score was 18.5 (IQR: 16.0–20.0) (Table 2).

**Table 2:** PrEP knowledge

	**Comparison**		**Intervention**		**Difference in difference** [Change (intervention)—Change (comparison)] ^c^*Adjusted for first ANC*		
**Indicator**	Pre (*N* = 420)	Post (*N* = 416)	Pre (*N* = 392)	Post (*N* = 408)	Point estimate	95% CI	*p*-value
HIV prevention	197 (46.9%)	165 (39.7%)	158 (40.3%)	187 (45.8%)	12.7%	(3.1%—22.2%)	0.009
Frequency of use	108 (25.7%)	72 (17.3%)	76 (19.4%)	95 (23.3%)	12.3%	(4.4%—20.2%)	0.002
Time to protection	34 (8.1%)	22 (5.3%)	26 (6.6%)	31 (7.6%)	3.8%	(−1.2%—8.7%)	0.134
Condom use	88 (21.0%)	48 (11.5%)	72 (18.4%)	78 (19.1%)	10.2%	(2.9%—17.5%)	0.006
Side effects	18 (4.3%)	7 (1.7%)	19 (4.8%)	17 (4.2%)	2.0%	(−1.6%—5.6%)	0.281
Discontinuation	30 (7.1%)	7 (1.7%)	16 (4.1%)	10 (2.5%)	3.8%	(0.1%—7.6%)	0.042

### Service and client outcomes

Among intervention and comparison facilities in our baseline assessments, waiting and service time ranged from 9–78.5 and 8–27 min, respectively (Supplementary Table 3). The implementation strategy bundle was not associated with changes in service and client outcomes. The client waiting time decreased non-significantly by 0.03 min (95%CI: −0.26–0.20 min; *p* = 0.796) in intervention sites vs comparison sites while there were no differences in service time (95%CI: −0.09–0.35 min; *p* = 0.249). Client satisfaction with services offered at the facility did not change substantially (−0.04/24 points; 95%CI: −0.40–0.33 min; *p* = 0.849) (Tables 1 and 2, Fig. 2).

### Secondary outcomes

We assessed the effect of the strategy bundle on different components of PrEP knowledge among women. These included: PrEP for HIV prevention, frequency of use, time to reach maximum protection, condom use while on PrEP, side effects, and discontinuation. The strategy bundle was associated with significant increases in several knowledge questions, despite the magnitude of the increase being heterogeneous. There were significant increase in: PrEP for HIV prevention (12.7%; 95%CI: 3.1%−22.2%; *p* = 0.009), frequency of PrEP use (12.3%; 95%CI: 4.4%−20.2%; *p* = 0.002), concurrent condom while on PrEP (10.2%; 95%CI: 2.9%−17.5%; *p* = 0.006), and PrEP discontinuation (3.8%; 95%CI: 0.1%−7.6%; *p* = 0.042) in intervention sites vs comparison sites. The implementation strategy bundle was associated with a non-significant increase in PrEP knowledge on: the time it takes for PrEP medication to reach maximum protection (3.8%; 95%CI: −1.2%−8.7%; *p* = 0.134) and side effects knowledge (2.0%; 95%CI: −1.6%−5.6%; *p* = 0.281) in intervention vs comparison sites. Overall, the strategy bundle was associated with a non-significant increase of 1.2% percentage points in correctly answering all PrEP knowledge questions (95%CI: −0.5%−2.8%; *p* = 0.181) in intervention vs comparison sites (Table 3, Fig. 2).

**Table 3:** Satisfaction, acceptability, appropriateness

	Comparison sites		Intervention sites	
	Pre (*N* = 480)	Post (*N* = 479)	Pre (*N* = 479)	Post (*N* = 479)
	Mean (SD)	Mean (SD)	Mean (SD)	Mean (SD)
Client satisfaction				
Quality of the service^a^	3.18 (0.53)	3.10 (0.42)	3.13 (0.56)	3.13 (0.47)
Got kind of service client wanted^b^	3.61 (0.51)	3.57 (0.51)	3.60 (0.58)	3.53 (0.55)
Extent to which this facility met your needs^c^	3.47 (0.52)	3.40 (0.50)	3.47 (0.59)	3.42 (0.54)
Would recommend friend to this facility^b^	3.85 (0.37)	3.87 (0.34)	3.86 (0.39)	3.92 (0.31)
Satisfied with the amount of help received^d^	3.36 (0.64)	3.27 (0.56)	3.32 (0.72)	3.18 (0.62)
Would come back to the facility^b^	3.89 (0.33)	3.90 (0.30)	3.90 (0.72)	3.93 (0.30)
Overall (out of 24 points)	21.36 (1.91)	21.10 (1.67)	21.27 (2.22)	21.12 (1.84)
HCW perceptions of appropriateness and acceptability of implementation strategy bundle				Post (*N* = 64)
				Mean (SD)
Appropriateness (IAM)^e^				
Fitting	–	–	–	4.48 (0.80)
Suitable	–	–	–	4.48 (0.67)
Applicable	–	–	–	4.42 (0.73)
A good match	–	–	–	4.57 (0.66)
Acceptability (AIM)^f^				
Meets approval	–	–	–	4.51(0.56)
Appealing	–	–	–	4.45 (0.59)
I like it	–	–	–	4.56 (0.50)
I welcome it	–	–	–	4.63 (0.49)

^a^Likert scale options: poor to excellent: 1–4

^b^Likert scale options: no, definitely not to yes, definitely: 1–4

^c^Likert scale options: none of my needs have been met to almost all of my needs have been met: 1–4

^d^Likert scale options: not satisfied to very satisfied: 1–4

^e^Average on 4 item Intervention Appropriateness Measure (IAM) scale; Likert scale (disagree to agree: 1–5)

^f^Average on 4 item Acceptability of Intervention Measures (AIM) scale; Likert scale (disagree to agree: 1–5)

### Sensitivity and stratified analyses

In analyses stratified by whether women were attending their first ANC visit versus any other visit type, we observed different effect sizes between the strata (Supplementary Table 4). Overall, the implementation strategy bundle was associated with larger increases in PrEP fidelity, PrEP risk screening, PrEP penetration, and PrEP offer among women receiving a visit other than their first ANC visit. HIV testing decreases associated with the implementation strategy bundle were more pronounced among women seeking their first ANC visit. Differences in client satisfaction and knowledge did not differ between strata. However, despite these larger relative differences for some outcomes, the absolute coverage levels for HIV testing and HIV risk screening were substantially higher among women attending their first ANC visit versus any other visit type (Supplementary Table 4).

In analyses stratified by whether a site experienced extended stockouts of HIV test kits (6 sites) versus no stockouts (2 sites), we observed that nearly all of the improvements associated with the implementation strategy bundle were observed in the sites without stockouts (Supplementary Table 5). The magnitude of the differences in PrEP fidelity, HIV testing, PrEP risk screening, PrEP penetration, PrEP offer, and client satisfaction were all larger in sites without stockouts versus sites without stockouts (Supplementary Table 5).

### Hypothetical best possible performance

As previously described [20], we calculated the hypothetical best performance of integrated PrEP delivery within MCH in the absence of modifying PrEP acceptance. We estimated the number of women who would hypothetically accept PrEP if PrEP counseling, risk assessment, HIV testing, and PrEP offer were perfectly delivered, with no changes in the proportion accepting PrEP when offered. Of the 1,636 women accessing care in MCH, we would expect 301 (31%) to be eligible for HIV testing, of whom 298 (99%) would test HIV negative, of whom 42 (14%) would accept PrEP if offered. Compared to the 9 women we observed to have initiated PrEP, 42 women represents a potential 4.6-fold increase in possible PrEP initiations if upstream implementation steps were perfected without modifying PrEP initiation.

## Discussion

In this study, prolonged stockouts of HIV testing commodities were associated with substantial negative impacts on service delivery during the study period. Overall, this stakeholder-selected implementation strategy package – fast-tracking PrEP refill clients in MCH, retraining providers, and PrEP dispensing in MCH clinic – was associated with enhanced PrEP penetration and PrEP offer and no changes in waiting or service time. The package was not associated with significant improvement in fidelity, HIV testing, or client satisfaction. This effect was most pronounced among sites that did not experience stockouts and among clients not seeking their first ANC visit. Despite substantial relative increases in implementation outcomes, large gaps persisted in absolute coverage.

There has been an expanding focus on both discovery science [4] and implementation science [21, 28] focused on PrEP for pregnant and postpartum populations in the past 5 years. Several trials, implementation projects, and qualitative projects in Kenya and South Africa have innovated key lessons on how PrEP integrated within MCH clinics can best be delivered. They have determined that integrated delivery of PrEP in MCH is feasible and acceptable [8, 15, 23], that PrEP delivery can be offered universally without a risk-guided approach [11], that video education and PrEP dispensing in MCH clinics are promising [20], that standardized patient actor training can provide high-quality education for providers [29]. Ongoing studies test stepped care to enhance continuation and persistence on PrEP during pregnancy after observations that discontinuation is high [30–32]. As new formulations of PrEP – including the vaginal ring and long-acting injectables – are implemented, many of these key implementation lessons are durable and can aid accelerated scale up of these new products to offer choices.

This study was the second in a planned set of four studies of implementation strategy bundle testing by a single team in a particular region. All four tests will use the same comparison facilities, partially aiding direct comparison. In the first test of video information, HIV self-testing, and PrEP dispensing in MCH [20], we observed comparable changes in PrEP penetration (5.4% percentage point increase in the prior study vs 6.0% percentage point increase in this study) and PrEP offer (4.4% vs 5.8%, respectively). We observed a larger impact on PrEP fidelity (7.6% vs 4.1%, respectively) and a smaller impact on PrEP screening (−8.8% vs 13.2%, respectively). Neither test had substantial impacts on waiting or service delivery time. The first test was associated with significantly improved client satisfaction, unlike this test, and the first test reflected slightly higher HCW acceptability and appropriateness scores compared to this second test. Despite the relative improvements in implementation outcomes, there remained large overall gaps in most implementation outcomes measured in both tests. Besides HIV testing, risk screening for PrEP had the highest coverage in both tests, reaching 32% and 36%, respectively, in intervention sites during the intervention period. The highest levels of the other implementation outcomes were more modest during this test compared to the first, likely due to stockout related interruptions in service delivery. In both tests, the optimization analysis revealed that if PrEP fidelity and offer were perfectly completed, several fold more women would have likely initiated PrEP (12-fold in test 1, 5.6-fold in this test), presenting remaining opportunities for upstream improvement in PrEP delivery.

We observed significant and moderate improvements in client PrEP knowledge in this study. In the first test, we observed that video education was associated with significant and larger improvements in client PrEP knowledge [20]. It is possible that improving client knowledge is better achieved through directly educating clients rather than providers or that the style of soap opera video was more effective at engaging and imparting knowledge to clients. A systematic review found that video education was superior to counselor-delivered information for HIV testing uptake [33] and a study in Kenya observed greater knowledge from HIV testing videos than counselor-delivered information [34]. Alternatively, the background temporal trends during this time period may have underestimated the true effect of provider training, resulting in an artificial difference. We were limited in that we were not able to assess knowledge among providers during this test to determine how effective the training was at imparting knowledge.

We observed that fast tracking as a strategy was more challenging to implement in practice, despite being raised as a feasible and effective strategy in formative qualitative [23] and quantitative prioritization [22] work. Fast tracking has been studied within differentiated HIV treatment [35] and PrEP delivery in HIV care clinics [36]; it is typically operationalized as both skipping some steps of a full visit, such as clinical review, and jumping to the beginning of a queue. In PrEP dispensing, fast tracking, in combination with other strategies, has been associated with shorter waiting times [36]. In HIV treatment, fast tracking has been associated with shorter waiting times than other forms of differentiated service models [35]. Within our study, it was not deemed acceptable by clinical teams for clients interested in PrEP to skip the queue at a general MCH clinic for perceptions of fairness. However, it was acceptable to mirror HIV treatment programs and fast track clients seeking PrEP refills. Given that the number of clients refilling PrEP was a small minority of overall clients in our study, it is unlikely that this strategy had a major impact on waiting time or service time for the overall population. However, for clients continuing PrEP, this may have had a benefit of promoting PrEP persistence, which has been a documented challenge in PrEP programs [32], similar to the benefits of fast tracking HIV treatment for retention [37].

This study tested a set of stakeholder-determined implementation strategies to improve delivery of PrEP in the absence of additional HCW. While not an intended aim of the study, it also assessed the impact of these strategies in the presence of widespread stockouts of HIV test kits. It is notable that the strategy bundle was only associated with improvements among sites *without* stockouts, suggesting that the results may not be durable in the face of commodity shortages, which are common in many resource-limited settings. Additionally, the differences in the impact of the strategy bundle between women seeking their first ANC visit versus any other type of visit suggests that this bundle may be most impactful during visit types where coverage of PrEP steps is already low, presenting greater room for improvement. The high uptake and prioritization of HIV testing among women attending their first visit aligns with national guidelines, which recommend routine testing at the initial visit unless HIV status is already known. The quasi-experimental design utilized improved our inference over a simple pre-post design, which would have failed to detect any impact.

Our study had several limitations. As previously mentioned, broad stockouts of HIV test kits occurred during the intervention period; however, this disruption likely resulted in an underestimate of the impact of the implementation strategy bundle, rather than overestimate. We were not able to collect process data on fast tracking or training, limiting our ability to precisely link these strategies to outcome changes. Fast-tracking likely had the most impact on PrEP continuation, which was not measured, rather than upstream steps, which were measured in this study. As mentioned during our team’s first test of an implementation strategy bundle [20], we were unable to measure clinical outcomes because of limitations with routine data sources differentiating PrEP clients who were initiated in MCH versus other venues in the facility. Additionally, we had originally aimed to use a controlled interrupted time series analysis, which has better control for temporal trends than a difference-in-differences design and would have allowed for a quantitative evaluation assessing the parallel trends assumption, but disruptions in services necessitated the switch. We allocated facilities to intervention vs comparison conditions prior to measuring baseline values, which would have afforded a better match distributions of baseline characteristics. Finally, the sites chosen for this study may be better resourced by virtue of having been previously selected for engagement in research studies [11, 13, 38]. Taken together, these limitations suggest that this pilot study is reasonable for identifying promising strategies with large effect sizes that could be further tested in a larger and more diverse sample of clinics to improve PrEP delivery in MCH.

## Conclusions

Fast-tracking, retraining providers on PrEP, and PrEP dispensing in an MCH clinic – a bundle of implementation strategies selected by stakeholders – improved some implementation outcomes for PrEP within MCH clinics; however, improvements were most pronounced in sites without sustained stockouts. PrEP penetration, PrEP offer, PrEP knowledge, and PrEP screening were significantly improved without changes to service time or waiting time. PrEP fidelity, client satisfaction, and HIV testing were not significantly impacted. This package, focused on retraining, co-delivery, and fast-tracking without additional HCW merits broader testing in diverse contexts. Persistent challenges in overall coverage remain, both during seasons with and without commodity stockouts.

## Supplementary Information

Supplementary Material 1.

Supplementary Material 2.

## Data Availability

The datasets used and/or analyzed during the current study are available from the corresponding author on reasonable request.
