# Supplementary material for: Impact of a stakeholder selected implementation strategy package – fast tracking, provider re-training, and co-location – on PrEP implementation for pregnant women in antenatal care clinics in western Kenya
Source: Implement Sci Commun. 2025 May 12;6:58. doi: 10.1186/s43058-025-00746-5 (PMC12067655; doi:10.1186/s43058-025-00746-5)
Supplement: Supplementary file 1 — Supplementary Material 1. [file 43058_2025_746_MOESM1_ESM.docx]

**Supplemental Tables & Figures:**

**Supplementary Table 1:** Difference in differences comparison of implementation, effectiveness, and service outcomes stratified by visit type (first ANC clients vs any other visit type)

|  | **Comparison sites** | | | | **Intervention sites** | | | | **Difference in difference** [(Change in intervention sites) – (Change in comparison sites)] **among first ANC clients** | | |  | **Comparison sites** | | | | **Intervention sites** | | | | **Difference in difference** [(Change in intervention sites) – (Change in comparison sites)] **among other visit type clients** | | |
| --- | --- | --- | --- | --- | --- | --- | --- | --- | --- | --- | --- | --- | --- | --- | --- | --- | --- | --- | --- | --- | --- | --- | --- |
| ***Outcome*** | Pre (N=84) | | Post (N=55) | | Pre (N=70) | | Post (N=51) | | Point estimate | Confidence interval | p-value |  | Pre (N=336) | | Post (N=361) | | Pre (N=322) | | Post (N=357) | | Point estimate | Confidence interval | p-value |
|  | N | n (%) or median IQR | N | n (%) or median IQR | N | n (%) or median IQR | N | n (%) or median IQR |  |  |  |  | N | n (%) or median IQR | N | n (%) or median IQR | N | n (%) or median IQR | N | n (%) or median IQR |  |  |  |
| **PrEP fidelity ^1^** | 71 | 5 (7.0%) | 48 | 1 (2.1%) | 60 | 8 (13.3%) | 39 | 1 (2.6%) | -5.81% | (-19.4% - 7.8%) | 0.401 |  | 92 | 3 (3.3%) | 111 | 1 (0.9%) | 119 | 1 (0.8%) | 109 | 8 (7.3%) | 8.60% | (2.2% - 15.0%) | 0.009 |
| HIV testing ^3^ | 72 | 65 (90.3%) | 48 | 39 (81.3%) | 60 | 52 (86.7%) | 41 | 24 (58.5%) | -22.20% | (-41.5% - 2.9%) | 0.024 |  | 92 | 29 (31.5%) | 111 | 31 (27.9%) | 120 | 41 (34.2%) | 109 | 39 (35.8%) | -1.90% | (-17.8% - 14.1%) | 0.817 |
| PrEP risk screening ^3^ | 84 | 48 (57.1%) | 55 | 32 (58.2%) | 70 | 42 (60.0%) | 51 | 37 (72.5%) | 11.50% | (-12.5% - 35.5%) | 0.347 |  | 336 | 53 (15.8%) | 361 | 55 (15.2%) | 322 | 55 (17.1%) | 357 | 109 (30.5%) | 14.20% | (6.0% - 22.3%) | 0.001 |
| **PrEP penetration ^1^** | 84 | 5 (5.9%) | 55 | 1 (1.8%) | 69 | 8 (11.6%) | 51 | 3 (5.9%) | -1.60% | (-13.8% - 10.6%) | 0.8 |  | 336 | 18 (5.4%) | 361 | 6 (1.7%) | 322 | 5 (1.6%) | 357 | 20 (5.6%) | 7.80% | (3.9% - 11.7%) | <0.001 |
| PrEP offer ^3^ | 84 | 5 (5.9%) | 55 | 0 (0%) | 70 | 6 (8.6%) | 51 | 2 (3.9%) | 1.4% | (-9.4% - 12.1%) | 0.802 |  | 336 | 19 (5.7%) | 360 | 7 (1.9%) | 322 | 6 (1.9%) | 357 | 18 (5.0%) | 6.9% | (3.0% - 10.9%) | 0.001 |
|  | 30 | 2 (6.7%) | 17 | 0 (0%) | 30 | 6 (20.0%) | 9 | 1 (11.1%) | -- | -- | -- |  | 12 | 2 (16.7%) | 14 | 1 (7.1%) | 9 | 1 (11.1%) | 14 | 7 (50.0%) | 48.4% | (5.4% - 91.4%) | 0.027 |
| PrEP uptake ^2^ | 5 | 0 (0%) | 0 | 0 (0%) | 6 | 2 (33%) | 2 | 0 (0%) | -- | -- | -- |  | 19 | 2 (10.5%) | 7 | 3 (42.9%) | 6 | 0 (0%) | 18 | 2 (11.1%) | -- | -- | -- |
|  | 84 | 0 (0%) | 55 | 0 (0%) | 70 | 2 (2.9%) | 51 | 0 (0%) | -- | -- | -- |  | 336 | 2 (0.6%) | 361 | 3 (0.8%) | 322 | 0 (0%) | 327 | 2 (0.6%) | -- | -- | -- |
| PrEP continuation ^2^ | 5 | 0 (0%) | 0 | 0 (0%) | 6 | 0 (0%) | 2 | 2 (100%) | -- | -- | -- |  | 19 | 6 (31.6%) | 7 | 1 (14.3%) | 6 | 0 (0%) | 18 | 2 (11.1%) | -- | -- | -- |
|  | 84 | 1 (1.2%) | 55 | 0 (0%) | 70 | 0 (0%) | 51 | 2 (3.9%) | -- | -- | -- |  | 336 | 6 (1.8%) | 361 | 3 (0.8%) | 322 | 7 (2.2%) | 327 | 6 (1.7%) | -- | -- | -- |
| **Client satisfaction ^1^** | 84 | 21.0 (20.0, 23.0) | 55 | 20.0 (20.0, 22.0) | 70 | 22.0 (20.0, 23.0) | 51 | 21.0 (20.0, 22.0) | 0.18 | (-0.88 - 1.23) | 0.741 |  | 336 | 22.0 (20.0, 23.0) | 361 | 21.0 (20.0, 22.0) | 322 | 22.0 (20.0, 23.0) | 357 | 21.0 (20.0, 22.0) | -0.06 | 0.78 | 0.622 |
| Client PrEP knowledge ^2^ | 84 | 1.2% | 55 | 0.0% | 70 | 0.0% | 51 | 0.0% | 1.20% | (-1.9% - 4.2%) | 0.445 |  | 336 | 1.5% | 361 | 0.3% | 322 | 0.9% | 357 | 0.8% | 1.10% | (-0.8% - 3.1%) | 0.258 |
|  | 84 | 1.13 | 55 | 0.67 | 70 | 1.14 | 51 | 1.14 | 0.45 | (-0.23 - 1.13) | 0.192 |  | 336 | 1.13 | 361 | 0.79 | 322 | 0.89 | 357 | 1.01 | 0.46 | (0.18 - 0.75) | 0.002 |
| *1: Primary outcome; 2: secondary outcome; 3: post hoc outcome* | | | | | | | | | | | |  |  |  | | | | | | | | | |

**Supplementary Table 2:** Demographic characteristics

|  |  | Comparison sites | | Intervention sites | |
| --- | --- | --- | --- | --- | --- |
|  | Overall (n=1,636) | Pre (n=420) | Post (n=416) | Pre (n=392) | Post (408) |
|  | n (%) or Median (IQR) | | | | |
| Age | 25.0 (21.0, 29.0) | 25.0 (22.0, 29.0) | 25.0 (22.0, 29.0) | 24.0 (21.0, 28.0) | 24.0 (21.0, 28.0) |
| First ANC | 260 (15.9%) | 84 (20.0%) | 55 (13.2%) | 70 (17.9%) | 51 (12.5%) |
| First ANC tested | 180 (69.2%) | 65 (77%) | 39 (71%) | 52 (74%) | 24 (47%) |

**Supplementary Table 3**: Site specific indicators by pre- post- period

| **Pre-period** | **Site 1** | **Site 2** | **Site 3** | **Site 4** | **Site 5** | **Site 6** | **Site 7** | **Site 8** | **Range** |
| --- | --- | --- | --- | --- | --- | --- | --- | --- | --- |
| Fidelity | 3.8% | 0.0% | 0.0% | 15.8% | 4.2% | 7.3% | 2.8% | 5.0% | 0-15.8% |
| Penetration | 3.6% | 1.0% | 1.9% | 15.7% | 3.0% | 3.7% | 3.2% | 3.3% | 1.0-15.7% |
| PrEP offer | 4.5% | 1.0% | 1.9% | 15.7% | 3.0% | 3.7% | 3.2% | 2.2% | 1.0-15.7% |
| HIV testing | 67.9% | 43.3% | 41.9% | 71.1% | 35.4% | 40.0% | 81.1% | 60.0% | 40.0-71.1% |
| Service time | 12 | 14 | 11.5 | 19 | 8 | 21 | 27 | 14 | 8-27 |
| Waiting time | 37.5 | 9 | 38.5 | 68 | 78.5 | 45 | 27.5 | 34 | 9-78.5 |
|  |  |  |  |  |  |  |  |  |  |
| **Post-period** | **Site 1** | **Site 2** | **Site 3** | **Site 4** | **Site 5** | **Site 6** | **Site 7** | **Site 8** | **Range** |
| Fidelity | 0.0% | 0.0% | 2.1% | 4.6% | 0.0% | 6.5% | 11.6% | 5.9% | 0-11.6% |
| Penetration | 2.8% | 2.0% | 0.9% | 1.0% | 3.0% | 6.5% | 5.5% | 7.7% | 0.9-7.7% |
| PrEP offer | 0.9% | 2.0% | 1.9% | 2.1% | 3.0% | 4.7% | 5.5% | 6.6% | 0.9-6.6% |
| HIV testing | 54.8% | 31.9% | 41.7% | 54.6% | 10.0% | 37.5% | 88.4% | 25.7% | 10.0-54.8% |
| Service time | 10.5 | 12.5 | 12 | 16 | 10 | 14 | 26 | 14.5 | 10-26 |
| Waiting time | 35 | 11.5 | 46.5 | 79 | 53 | 41.5 | 49.5 | 28 | 28-79 |

**Supplementary Table 4:** Site specific details

| **Facility** | **Level** | **County** | **Volume (High vs Low)*** |
| --- | --- | --- | --- |
| Site 1** | Sub county | Siaya County | High Volume |
| Site 2** | Sub county | Homabay county | High Volume |
| Site 3 | Sub county | Kisumu County | High Volume |
| Site 4** | Sub county | Siaya County | High Volume |
| Site 5** | Health center | Siaya County | Low Volume |
| Site 6** | Sub county | Siaya County | High Volume |
| Site 7** | Health center | Homabay county | Low Volume |
| Site 8 | Sub county | Kisumu County | High Volume |

**We used a cut off of 3000 antenatal clients per month to distinguish between high and low volume sites*

***Site previously engaged in a research trial*

**Supplementary Table 5:** Difference in differences comparison of implementation, effectiveness, and service outcomes stratified by HIV stockouts (Sites without stockouts vs Sites with stockouts)

|  | Sites without stockouts (N=2) | | | | | | | | | | |  | Sites with stockouts (N=6) | | | | | | | | | | |
| --- | --- | --- | --- | --- | --- | --- | --- | --- | --- | --- | --- | --- | --- | --- | --- | --- | --- | --- | --- | --- | --- | --- | --- |
|  | **Comparison sites** | | | | **Intervention sites** | | | | **Difference in difference [(Change in intervention sites) – (Change in comparison sites)] among sites without stockouts** | | |  | **Comparison sites** | | | | **Intervention sites** | | | | **Difference in difference [(Change in intervention sites) – (Change in comparison sites)] among sites with stockouts** | | |
|  | Pre (N=102) | | Post (N=97) | | Pre (N=94) | | Post (N=110) | | Point estimate | Confidence interval | p-value |  | Pre (N=318) | | Post (N=319) | | Pre (N=298) | | Post (N=298) | | Point estimate | Confidence interval | p-value |
| ***Outcome*** | N | n (%) or median IQR | N | n (%) or median IQR | N | n (%) or median IQR | N | n (%) or median IQR |  |  |  |  | N | n (%) or median IQR | N | n (%) or median IQR | N | n (%) or median IQR | N | n (%) or median IQR |  |  |  |
| **PrEP fidelity 1** | 38 | 6 (15.8%) | 22 | 1 (4.6%) | 36 | 1 (2.8%) | 43 | 5 (11.6%) | 20.15% | (0.4% - 39.9%) | 0.046 |  | 125 | 2 (1.6%) | 137 | 1 (0.7%) | 143 | 8 (5.6%) | 105 | 4 (3.8%) | -1.07% | (-6.9% - 4.8%) | 0.719 |
| HIV testing 3 | 38 | 27 (71.1%) | 22 | 12 (54.6%) | 37 | 30 (81.1%) | 43 | 38 (88.4%) | 16.60% | (-8.8% - 42.0%) | 0.199 |  | 126 | 67 (53.2%) | 137 | 58 (42.3%) | 143 | 63 (44.1%) | 107 | 25 (23.4%) | -13.90% | (-28.2% - 4.7%) | 0.058 |
|  | 25 | 21 (84.0%) | 7 | 5 (71.4%) | 14 | 12 (85.7%) | 11 | 10 (90.9%) | -- | -- | -- |  | 59 | 44 (74.6%) | 48 | 34 (70.8%) | 56 | 40 (71.4%) | 40 | 14 (35.0%) | -- | -- | -- |
| PrEP risk screening 3 | 102 | 32 (31.4%) | 97 | 30 (30.9%) | 94 | 19 (20.2%) | 110 | 50 (45.5%) | 20.70% | (3.4% - 37.9%) | 0.019 |  | 318 | 69 (21.7%) | 319 | 57 (17.9%) | 298 | 78 (26.2%) | 298 | 96 (32.2%) | 10.50% | (1.8% - 19.2%) | 0.018 |
| **PrEP penetration 1** | 102 | 16 (15.7%) | 97 | 1 (1.0%) | 93 | 3 (3.2%) | 110 | 6 (5.5%) | 16.89% | (7.5% - 26.3%) | <0.001 |  | 318 | 7 (2.2%) | 319 | 6 (1.9%) | 298 | 10 (3.4%) | 298 | 17 (5.7%) | 2.70% | (-1.2% - 6.7%) | 0.174 |
| PrEP offer 3 | 102 | 16 (15.7%) | 97 | 2 (2.1%) | 94 | 3 (3.2%) | 110 | 6 (5.5%) | 15.9% | (6.3% - 25.5%) | 0.001 |  | 317 | 8 (2.5%) | 318 | 5 (1.6%) | 298 | 9 (3.0%) | 298 | 14 (4.7%) | 2.70% | (-1.2% - 6.4%) | 0.166 |
|  | 5 | 2 (40%) | 5 | 1 (20%) | 5 | 1 (20%) | 9 | 5 (55.6%) | 51.80% | (-23.6% - 127.2%) | 0.178 |  | 37 | 2 (5.4%) | 26 | 0 (0%) | 34 | 6 (17.7%) | 14 | 3 (21.4%) | 9.1% | (-13.9% - 32.0%) | 0.439 |
| PrEP uptake 2 | 16 | 1 (6%) | 2 | 2 (100%) | 3 | 0 (0%) | 6 | 1 (16.7%) | -- | -- | -- |  | 8 | 1 (12.5) | 5 | 1 (20.0) | 9 | 2 (22.2%) | 14 | 1 (7.1%) | -- | -- | -- |
|  | 102 | 1 (1.0%) | 97 | 2 (2.1%) | 94 | 0 (0%) | 110 | 1 (0.9%) | -- | -- | -- |  | 318 | 1 (0.3%) | 318 | 1 (0.3%) | 298 | 2 (0.7%) | 298 | 1 (0.3%) | -- | -- | -- |
| PrEP continuation 2 | 16 | 4 (25.0%) | 2 | 0 (0%) | 3 | 2 (66.7%) | 6 | 1 (16.7%) | -- | -- | -- |  | 8 | 2 (25.0%) | 5 | 1 (20.0%) | 9 | 0 (0%) | 14 | 5 (35.7%) | -- | -- | -- |
|  | 102 | 4 (3.9%) | 97 | 0 (0%) | 94 | 3 (3.2%) | 110 | 2 (1.8%) | -- | -- | -- |  | 318 | 3 (0.9%) | 319 | 3 (0.9%) | 298 | 4 (1.3%) | 298 | 6 (2.0%) | -- | -- | -- |
| **Service time 1** | 48 | 19 (13 - 29) | 48 | 16 (10 - 23) | 48 | 27 (16.5 - 47.5) | 48 | 26 (15 - 43) | -- | -- | -- |  | 144 | 13 (9 - 25) | 144 | 12 (8 - 18) | 144 | 13 (8 - 24.5) | 144 | 13 (9 - 21.5) | -- | -- | -- |
| **Waiting time 1** | 48 | 68 (48 - 115) | 48 | 79 (55.5 - 111) | 48 | 27.5 (10.5 - 53.5) | 48 | 49.5 (33 - 81.5) | -- | -- | -- |  | 144 | 26 (10 - 47) | 144 | 28 (11 - 49.5) | 144 | 53.5 (27.5 - 85) | 144 | 40 (26 - 61.5) | -- | -- | -- |
| **Client satisfaction 1** | 102 | 22.0 (21.0, 23.0) | 97 | 22.0 (20.0, 22.0) | 94 | 22.5 (21.0, 23.0) | 110 | 22 (20.0, 23.0) | 0.05 | (-0.74 - 0.84) | 0.9 |  | 318 | 21.0 (20.0, 23.0) | 319 | 21.0 (20.0, 22.0) | 298 | 21.0 (20.0, 23.0) | 298 | 21.0 (20.0, 22.0) | -0.04 | (-0.45 - 0.36) | 0.834 |
| Client PrEP knowledge 2 | 102 | 2.0% | 97 | 0.0% | 94 | 0.0% | 110 | 0.0% | -- | -- | -- |  | 318 | 1.3% | 319 | 0.3% | 298 | 1.0% | 298 | 1.0% | 0.90% | (-1.2% - 3.0%) | 0.385 |
|  | 102 | 1.225 | 97 | 0.723 | 94 | 0.883 | 110 | 1.018 | 0.629 | (0.096- 1.162) | 0.021 |  | 318 | 1.102 | 319 | 0.787 | 298 | 0.953 | 298 | 1.027 | 0.39 | (0.09 - 0.69) | 0.012 |

** not adjusted for visit type due to visit type not collected during time-and-motion activity*

*1: Primary outcome; 2: secondary outcome; 3: post hoc outcome*
