# Supplementary material for: Impact of a stakeholder selected implementation strategy package – fast tracking, provider re-training, and co-location – on PrEP implementation for pregnant women in antenatal care clinics in western Kenya
Source: Implement Sci Commun. 2025 May 12;6:58. doi: 10.1186/s43058-025-00746-5 (PMC12067655; doi:10.1186/s43058-025-00746-5)
Supplement: Supplementary file 2 — Supplementary Material 2. [file 43058_2025_746_MOESM2_ESM.pdf]

Client Exit Survey

Record ID ______________________

Client exit survey PTID ______________________

CLIENT EXIT SURVEY PTID DOES NOT MATCH RECORD ID. VERIFY AND CORRECT ID FOR THIS CLIENT SO THAT BOTH IDS MATCH

PTID DOES NOT MATCH ALLOCATION FOR THIS DATA COLLECTION ID. REVIEW PTID ASSIGNMENT SOP AND REALLOCATE ID.

Timestamp ______________________

Is the client willing to participate? Remind them they are not forced to participate in any activities and may chose not to answer any question at any time.
○ Yes ○ No
(Follow the oral consent guide)

Date of Exit Survey ______________________

Section 1: Participant Characteristics

What is your age? ______________________
((in years))

Is this your first antenatal care (ANC) visit for your current pregnancy?
○ Yes ○ No

Were you tested for HIV at today's visit?
○ Yes ○ No

If you were not tested for HIV today, when were you last tested for HIV? ______________________
(If the client does not know the exact date they were last tested, choose the midpoint of the month (15th) or year if the month is unknown (July 1st))

Can I ask about your HIV status?
○ Yes ○ No

Do you know your HIV status?
○ Yes ○ No ○ Don't know

If yes, what is your HIV status?
○ HIV Negative ○ HIV Positive

Please answer 'yes' if you were asked about ANY of the following behaviors today:<br>a. number of sexual partners;<br>b. HIV status of sexual partners;<br>c. sex without a condom<br>c. signs or symptoms of a sexually transmitted infection;<br>d. recent diagnosis or treatment for a sexually transmitted infection;<br>e. sex in exchange for money or other favors;<br>f. being forced to have sex against your will;<br>g. being physically assaulted including assault by your sex partner;<br>h. sex while your partner or you were under the influence of alcohol or drugs;<br>i. sharing needles while engaging in intravenous drug use<br>j, using post exposure prophylaxis (PEP) more than twice?	◯ Yes ◯ No ◯ Don't know
Please answer 'yes' if ANY of the following statements are true about your experience within the past 6 months:<br>a. number of sexual partners (>1);<br>b. HIV status of sexual partners (HIV positive or unknown HIV status);<br>c. sex without a condom<br>c. signs or symptoms of a sexually transmitted infection;<br>d. recent diagnosis or treatment for a sexually transmitted infection;<br>e. sex in exchange for money or other favors;<br>f. being forced to have sex against your will;<br>g. being physically assaulted including assault by your sex partner;<br>h. sex while your partner or you were under the influence of alcohol or drugs;<br>i. sharing needles while engaging in intravenous drug use<br>j, using post exposure prophylaxis (PEP) more than twice?	◯ Yes ◯ No ◯ Don't know
Did anyone talk to you about PrEP today?	◯ Yes ◯ No ◯ Don't know
During your visit today, were you told that PrEP can lower your risk of becoming infected with HIV?	◯ Yes ◯ No ◯ Don't know
During your visit today, were you offered to start or continue taking PrEP?	◯ Yes<br>◯ No<br>◯ No, but if I had been offered, I would have been interested in starting or continuing PrEP<br>◯ No, and even if I had been offered, I would NOT have been interested in starting or continuing PrEP<br>◯ Don't know
During your visit today, did you decide to use PrEP?	◯ No ◯ I was already taking PrEP and will continue to take PrEP ◯ Yes, I initiated PrEP today

Section II: PrEP Knowledge Questions

PrEP is a medication that can be used to prevent which of the following?

	Yes	No	Don't know
Sexually Transmitted Infections (Other than HIV)	○	○	○
HIV	○	○	○
Pregnancy	○	○	○

How often should PrEP be taken?

	Yes	No	Don't know
Every day	○	○	○
Once a week	○	○	○
Whenever you have sex	○	○	○

How long does it take for PrEP to reach maximum protection?

	Yes	No	Don't know
Immediately	○	○	○
3 days	○	○	○
7 days	○	○	○
28 days	○	○	○

When you are taking PrEP, you do not need to use condoms. ○ True ○ False ○ Don't know

Which of the following are common side effects when starting PrEP?

	Yes	No	Don't know
Nausea	○	○	○
Dizziness	○	○	○
Headache	○	○	○
Fatigue	○	○	○

PrEP should be discontinued when:

	Yes	No	Don't know
A week after a person stops being at risk for acquiring HIV	○	○	○
A month after a person stops being at risk for acquiring HIV	○	○	○
A woman becomes pregnant	○	○	○
A women starts breastfeeding	○	○	○
A person becomes HIV-positive	○	○	○
Your partner tests HIV-positive	○	○	○

Section III: Please help improve this facility by answering some questions about the services you received at this facility today. We are interested in your honest opinions, whether they are positive or negative. Your answers will remain private. Please answer all of the questions. We also welcome you comments and suggestions. Thank you very much, we appreciate your help.

How would you rate the quality of the service you received?	◯ Excellent ◯ Good ◯ Fair ◯ Poor
Did you get the kind of service you wanted?	◯ No, Definitely not ◯ No, not really ◯ Yes, generally ◯ Yes, Definitely
To what extent has this facility met your needs?	◯ Almost all of my needs have been met<br>◯ Most of my needs have been met<br>◯ Only a few of my needs have been met<br>◯ None of my needs have been met
If a friend were in need of similar help, would you recommend this facility to him/her?	◯ No, definitely not ◯ No, I don't think so ◯ Yes, I think so ◯ Yes, definitely
Were you satisfied with the amount of help you received?	◯ Unsatisfied ◯ Indifferent or mildly satisfied ◯ Mostly satisfied ◯ Very satisfied
If you were to seek help again, would you come back to this facility?	◯ No, definitely not ◯ No, I don't think so ◯ Yes, I think so ◯ Yes, definitely
Do you have any other comments or suggestions for us?	______________________________
